# Intensive insulin therapy does not alter the inflammatory response in patients undergoing coronary artery bypass grafting: a randomized controlled trial [ISRCTN95608630]

**DOI:** 10.1186/cc3911

**Published:** 2005-11-16

**Authors:** Cornelia W Hoedemaekers, Peter Pickkers, Mihai G Netea, Marcel van Deuren, Johannes G Van der Hoeven

**Affiliations:** 1 Department of Intensive Care, Radboud University Nijmegen Medical Centre, Geert Grooteplein 10, 6500 HB Nijmegen, The Netherlands; 2 Department of Internal Medicine, Radboud University Nijmegen Medical Centre, Geert Grooteplein 10, 6500 HB Nijmegen, The Netherlands

## Abstract

**Introduction:**

Strict control of plasma glucose in diabetic and non-diabetic patients has been shown to improve outcome in several clinical settings. There is extensive evidence that glucose can stimulate the production of pro-inflammatory cytokines such as tumor necrosis factor (TNF)-α and IL-6, with no effect on the anti-inflammatory cytokine IL-10. We hypothesized that strict glucose regulation results in a change in cytokine balance from a pro-inflammatory state to a more balanced anti-inflammatory condition. In a randomized controlled trial we studied the effect of strict glycemic control on the local and systemic pro-inflammatory and anti-inflammatory balance in non-diabetic patients undergoing elective coronary artery bypass grafting with cardiopulmonary bypass.

**Methods:**

After surgery patients were randomly assigned to intensive insulin therapy (blood glucose between 80 and 110 mg/dl) or conventional insulin therapy (blood glucose less than 200 mg/dl). At 0, 1, 2, 4, 8, 12, 16 and 24 hours after admission to the intensive care unit, plasma samples and samples from the mediastinal drains were obtained. We measured the concentrations of the pro-inflammatory cytokines TNF-α and IL-6 and the anti-inflammatory cytokine IL-10 by enzyme-linked immunosorbent assay.

**Results:**

Both patient groups were comparable in demographics, clinical characteristics and peri-operative data. In the intensive treatment group, glucose levels were significantly lower than in the conventionally treated group. No differences were found between both groups in the concentrations of TNF-α, IL-6 and IL-10 in plasma samples or in fluid draining the mediastinal cavity. Levels of IL-6 and IL-10 were significantly higher in mediastinal fluid samples than in plasma samples, suggesting a compartmentalized production of cytokines.

**Conclusion:**

The protective effect of intensive insulin therapy in patients after cardiac surgery with cardiopulmonary bypass is not related to a change in cytokine balance from a pro-inflammatory to an anti-inflammatory pattern. Systemic cytokine levels are not representative of the local inflammatory response.

## Introduction

Strict glycemic control is increasingly recognized as an important goal in a broad spectrum of critically ill patients, even in the absence of pre-existing diabetes. After myocardial infarction, stress hyperglycemia is associated with an increased risk of in-hospital mortality in both diabetic and non-diabetic patients and increases the risk of congestive heart failure or cardiogenic shock in patients without diabetes [1]. The DIGAMI study demonstrated that rapid improvement of metabolic control in diabetic patients with myocardial infarction improves short-term and long-term outcome [2,3]. Intensive insulin therapy is thought to increase the success rate of thrombolysis and preserves myocardial function [4,5]. The recently published DIGAMI 2 trial failed to support the hypothesis that insulin treatment improves survival and morbidity in diabetic patients after myocardial infarction, possibly because the target blood glucose levels in the treatment group were never reached [6]. However, this trial confirmed that glucose is a strong and independent factor of long-term mortality in these patients. In cardiac surgery patients, hyperglycemia was found to be an independent post-operative risk factor for the development of hyperlactatemia and associated with increased morbidity and mortality [7,8]. A meta-analysis of all randomized studies using glucose-insulin-potassium therapy in cardiac surgery patients indicated that such therapy might considerably improve post-operative recovery of contractile function and reduce the incidence of atrial arrhythmias [9]. Some studies even show a survival benefit [10]. In critically ill patients in a surgical intensive care unit (ICU), maintenance of blood glucose levels between 80 and 110 mg/dl resulted in a 42% reduction in mortality compared with conventional treatment aiming at blood glucose levels between 180 and 200 mg/dl [11]. More than 60% of the patients in this study had also recently had cardiac surgery.

How strict control of blood glucose reduces morbidity and mortality is unknown, but the mechanism may be related either to a direct effect of normalization of hyperglycemia or to the concomitantly higher insulin levels. *Post hoc* multivariate logistic regression analysis of the study by van den Berghe *et al*. suggests that the lowered blood glucose level rather than the insulin dose is related to the reduction in mortality [12,13]. In this study [11] septic patients showed the largest reduction in mortality, suggesting that strict glucose regulation might influence the inflammatory response.

Hyperglycemia has a strong impact on host defense. Acute, short-term hyperglycemia affects all major components of innate immunity [14]. Neutrophil activity is reduced, leading to decreased chemotaxis, decreased phagocytosis, decreased bacterial killing and overproduction of free radicals [15-17]. In addition to changes in cellular function, other components of the innate immune response contribute to the pro-inflammatory state in hyperglycemia. *In vitro*, human monocytes show a glucose-dependent increase in tumor necrosis factor (TNF)-α and IL-6 production [18-20]. Healthy volunteers have an increase in the pro-inflammatory cytokines IL-6, TNF-α and IL-18 when the plasma glucose level is acutely raised by glucose infusion while endogenous insulin secretion is blocked with octreotide [21]. Patients with hyperglycemia on admission to the ICU had increased levels of IL-6 and IL-10, although after multivariate analysis only IL-6 was associated with hyperglycemia [22].

Studies of cytokine responses in critically ill patients are potentially confounded by the absence of a well-defined time of onset and differences in the etiology and severity of the disease. Cardiac surgery is associated with the development of a systemic inflammatory reaction with increased cytokine concentrations. Inflammation after cardiac surgery is believed to be caused mainly by contact of blood with the artificial surface of the extracorporeal circuit, and also by ischemia-reperfusion injury, and the operative trauma [23,24]. It has been suggested that the release of pro-inflammatory cytokines into the circulation is important in the pathogenesis of post-operative myocardial dysfunction [25-28]. We studied the effect of strict glycemic control on the local and systemic pro-inflammatory and anti-inflammatory cytokine balance in cardiac surgery patients after cardiopulmonary bypass. We considered cardiac surgery with cardiopulmonary bypass to be a suitable clinical model of inflammation with a well-defined preset time of onset and etiology of the insult, in contrast to other clinical models of inflammation such as sepsis. We hypothesized that strict glucose regulation modulates cytokine production in these patients, leading to a shift toward a more anti-inflammatory pattern [29].

## Methods

### Study population

We performed a randomized controlled trial in non-diabetic patients undergoing elective coronary artery bypass grafting (CABG). The local Institutional Review Board approved the protocol. Written informed consent was obtained from each patient on the day before surgery. All patients aged 18 years or older scheduled for elective CABG were eligible for the study. Patients were excluded if they had a history of diabetes, fasting blood glucose levels above 100 mg/dl on the day before surgery, myocardial infarction within 4 weeks before surgery, cardiogenic shock or renal failure (serum creatinine level above 1.7 mg/dl). Patients were also excluded if they had used any medication within 4 weeks before surgery known to modulate the inflammatory response (for example non-steroidal anti-inflammatory drugs or steroids) or when there were clinical signs of infection or inflammatory disease. The use of low-dose salicylates was allowed. Patients undergoing off-pump cardiac surgery were excluded. During surgery no blood glucose concentrations were measured, and none of the patients received insulin before admission to the ICU. Cardiopulmonary bypass was performed with a priming solution containing gelatin (Gelofusine^®^), mannitol, albumin, NaHCO_3_, CaCl_2 _and heparin. After weaning from cardiopulmonary bypass, patients were given protamine to neutralize the heparin. Heparin antagonization was identical in both groups.

### Study design

Patients were randomly assigned to receive intensive or conventional treatment. Assignments to the treatment groups were made with the use of sealed envelopes. In the intensive treatment group, patients received insulin (Actrapid HM; Novo Nordisk, Copenhagen, Denmark) intravenously to maintain blood glucose levels between 80 and 110 mg/dl. In the conventional treatment group, insulin therapy was given when blood glucose levels exceeded 200 mg/dl. Similarly to the trial by van den Berghe *et al*. [11], treatment started immediately on admission to the ICU. Blood glucose levels were measured hourly and adjusted according to a nomogram based on the study by van den Berghe *et al*. [11]. On admission, all patients were infused continuously with 3.75 g of intravenous glucose per hour. Blood samples for the measurement of systemic cytokine concentration and drain samples from the mediastinal cavity (local cytokine release) were taken on arrival in the ICU and at 1, 2, 4, 8, 12, 16 and 24 hours after admission.

### Cytokine analysis

Blood and samples from the tubes draining the mediastinal cavity were immediately centrifuged for 15 minutes at 2,000 *g *at 4°C, and serum and supernatants were stored at -80°C until measurement in a single batch. Concentrations of TNF-α, IL-6 and IL-10 were measured in accordance with the manufacturer's instructions with a commercial sandwich-type enzyme-linked immunosorbent assay (PeliKine; Sanquin, Amsterdam, The Netherlands).

### Statistical analysis

Power calculation was based on clinically relevant changes in serum IL-6 levels. In previous studies a standard deviation of 14 to 16% of baseline values was found [30-32]. An insulin-mediated decrease of 15% in IL-6 or a 15% increase in IL-10 was considered to indicate a clinically relevant change in the pro-inflammatory and anti-inflammatory balance. With an estimated SD of 15% and a significance level *α *of 0.05, a sample size of nine patients per group was calculated to reach a power of 90%. We therefore included 10 patients per group in the present study. Changes in cytokine levels over time were analyzed with one-way analysis of variance. Differences between groups were analyzed with two-way analysis of variance. *p *< 0.05 was considered statistically significant. All data are expressed as median (interquartile range unless otherwise stated).

## Results

### Study population

A total of 20 patients were enrolled in the study. The clinical and demographic characteristics of the two groups at randomization are shown in Table 1. No differences were found with respect to age, body mass index, duration of operation or time on cardiopulmonary bypass. No major perioperative complications were reported. APACHE (Acute Physiology and Chronic Health Evaluation) II and Parsonnet scores were comparable on arrival in the ICU. Blood glucose levels on admission were slightly higher in the intensive treatment group than in the conventional treatment group (mean ± SD 114.4 ± 15.1 versus 97.6 ± 19.8 mg/dl; *p *= 0.05).

No major complications occurred during the post-operative stay in any of the patients. Post-operative time on the ventilator and the time in the ICU were comparable in both groups (Table 2). Both groups had similar increases in creatinine kinase levels as a global measure of tissue damage. Equal amounts of erythrocyte and platelet transfusions were administered in both treatment arms.

### Blood glucose control

**Figure 1:**
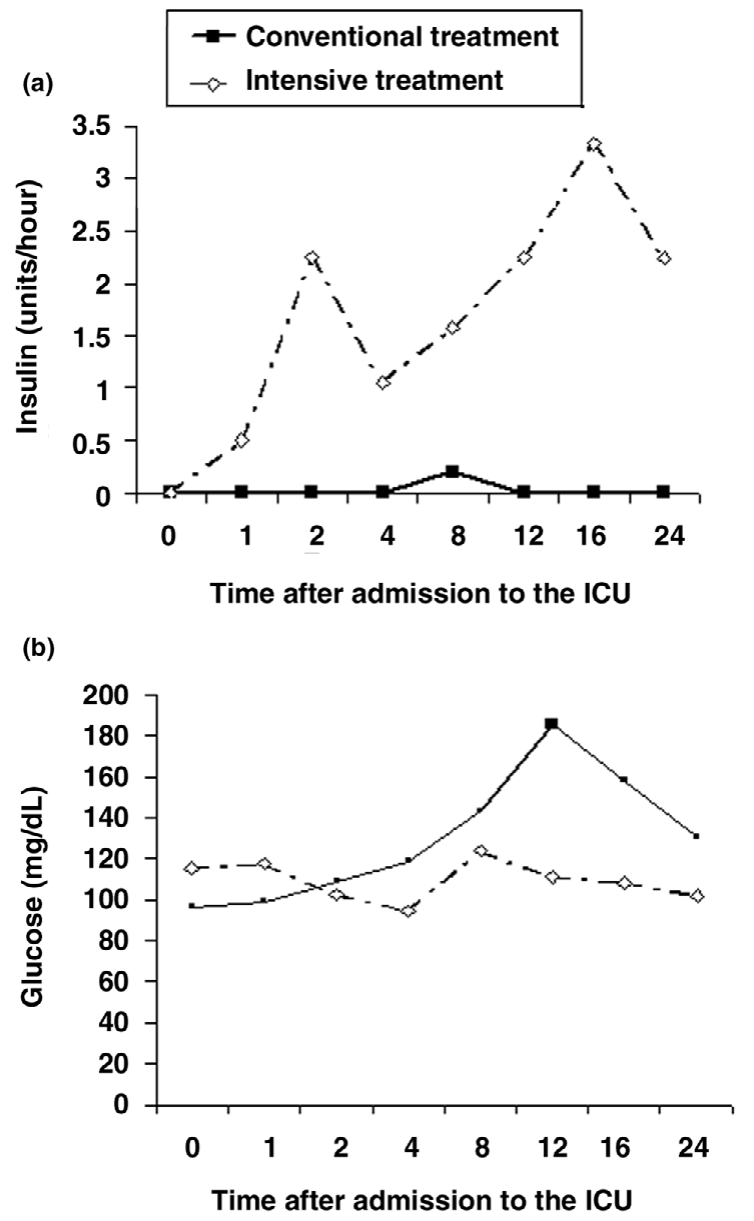
Insulin dosage and glucose concentration are significantly different between the treatment groups. Insulin dosage per hour **(a) **and glucose concentration in serum **(b) **in the intensive and conventional treatment group after admission to the intensive care unit. Points are median values.

In the intensive treatment group all patients required exogenous insulin, whereas in the conventionally treated group only one patient received a low dose of insulin when blood glucose exceeded 200 mg/dl (Figure 1a). Blood glucose levels were significantly lower in the intensive treatment group than in the conventional treatment group (*p *< 0.003) (Figure 1b). Hypoglycemia (defined as a blood glucose level of 40 mg/dl or less) did not occur in any of the patients. The rise in mean glucose levels in the placebo group, together with the escalating insulin dosage in the strict control group, suggests increasing insulin resistance after surgery. This may be explained by increasing catecholamine concentrations, because the increased glucose levels coincided with tapering of the anesthetic agents. Hemodynamic parameters such as blood pressure, heart rate, urine production and administration of inotropic agents were comparable between the groups (data not shown).

### TNF-α, IL-6 and IL-10 concentration in serum

**Figure 2:**
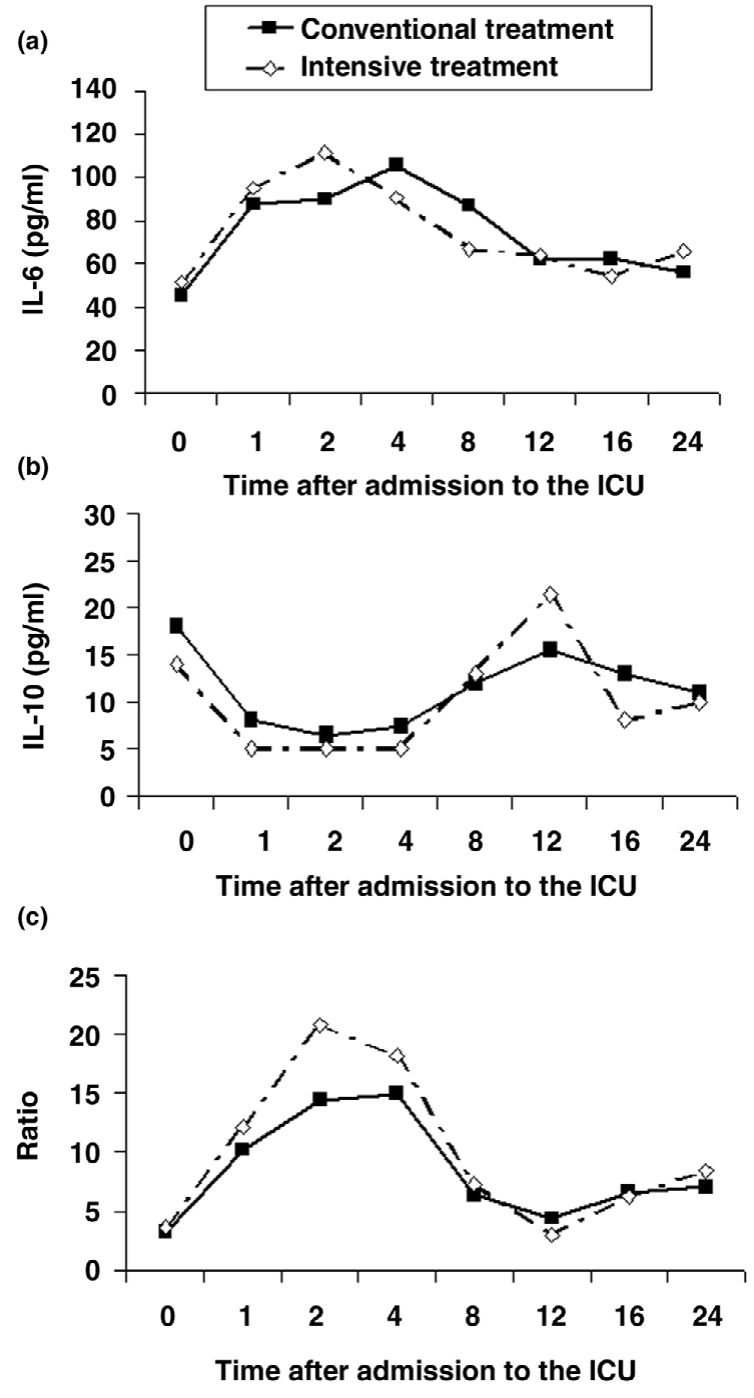
Systemic cytokine concentrations are comparable between the treatment groups. Concentration of IL-6 **(a) **and IL-10 **(b) **at different time points after admission to the intensive care unit. **(c) **The ratio of IL-6 to IL-10 in serum is expressed as arbitrary units. No significant differences are found between both treatment groups. Points are median values.

Systemic concentration of TNF-α did not change statistically during the first post-operative day (data not shown). IL-6 increased post-operatively with maximum values 2 to 4 hours after admission (*p *< 0.001), followed by a gradual decline (Figure 2a). The anti-inflammatory cytokine IL-10 was increased on admission and showed a second peak at 12 hours after admission (*p *= 0.002) (Figure 2b). No differences were found between the conventional and intensive treatment group with regard to the pattern and levels of systemic cytokine production. The ratio of IL-6 to IL-10 increased in the first hours with a maximum at 2 hours after admission (*p *< 0.001) and showed a rapid decline afterwards (Figure 2c).

### TNF-α, IL-6 and IL-10 concentration in samples from the mediastinal cavity

**Figure 3:**
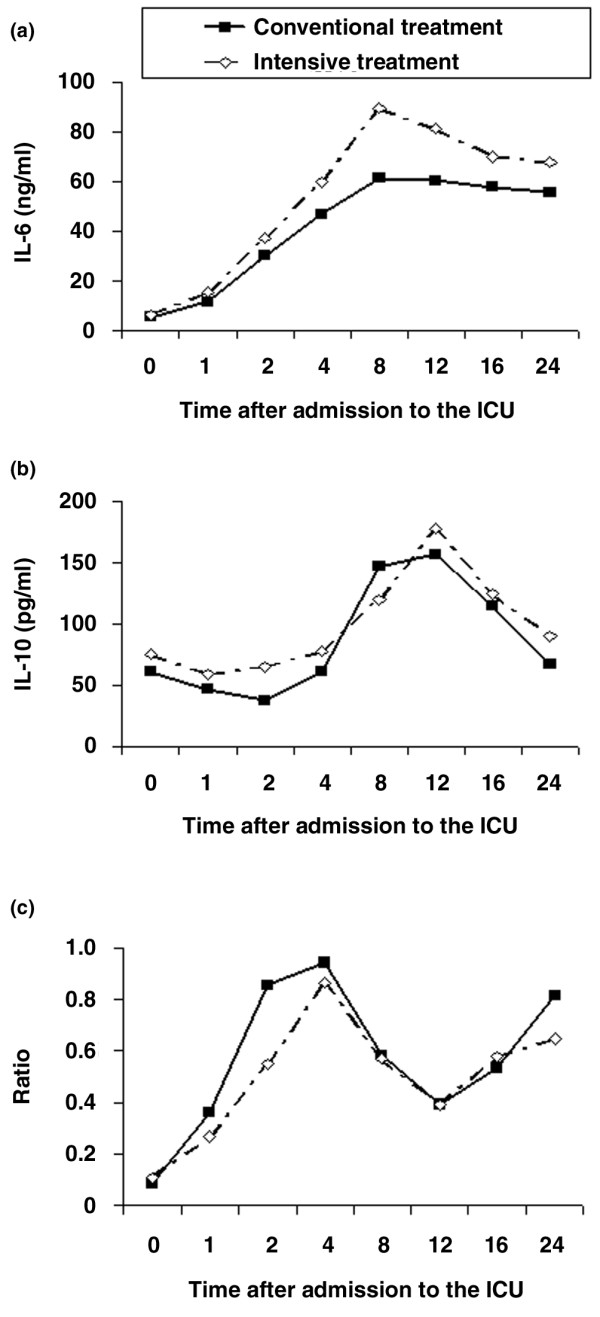
Cytokine production is highly compartmentalized. Concentration of IL-6 **(a) **and IL-10 **(b) **in mediastinal fluid at different time points after admission to the intensive care unit. **(c) **The ratio of IL-6 to IL-10 in mediastinal fluid is expressed as arbitrary units. No significant differences are found between both treatment groups. IL-6 and IL-10 levels are significantly higher in the mediastinal fluid samples than in plasma samples, indicating local production. Points are median values.

Cytokines were measured in fluids from the mediastinal drains. IL-6 levels from the mediastinal cavity increased and reached a maximum at 8 hours after admission (*p *< 0.001) (Figure 3a). Levels of IL-6 in the tubes draining the mediastinal cavity were about 1,000-fold higher than the values measured in blood. IL-10 concentrations showed a peak at 12 hours after admission (*p *< 0.001) and were about 10 times higher in the samples from the mediastinum than in the systemic levels (Figure 3b). No differences were found in the mediastinal levels of TNF-α, IL-6 and IL-10 between the treatment groups. The IL-6/IL-10 ratio had a peak at 4 hours after admission, followed by a second rise at 12 hours after admission (*p *< 0.001) (Figure 3c).

## Discussion

The main conclusion from the present study is that strict glucose regulation does not alter cytokine concentrations in the plasma and in the mediastinal cavity in patients after CABG. Strict glucose control also had no effect on activation of the terminal complement complex or leukocyte numbers (data not shown). These results suggest that the beneficial effect of intensive insulin therapy in patients after cardiac surgery is not mediated by changes in cytokine balance.

The beneficial effects of strict glucose control in the study by van den Berghe *et al*. [11] are supported by a study in diabetic patients undergoing cardiac surgery in which continuous insulin therapy reduced the risks of death and wound infections by 57% and 66%, respectively [33]. Multivariate logistic regression analysis has shown that the beneficial effects of strict glucose control in the study by van den Berghe *et al*. [11] was related mainly to an improvement in dyslipidemia [34]. However, this *post hoc* analysis included only patients admitted for 7 days or longer and is therefore clearly different from our uncomplicated CABG patients. It is nevertheless possible that the pro-inflammatory effects of increased fatty acid levels also have a role in acute illness. Infusion of triglycerides in healthy volunteers acutely increased NF-κB binding activity and p65 expression in circulating monocytes [35]. Activation of NF-κB induces an inflammatory response by the increased transcription of genes that are involved in the production of pro-inflammatory cytokines and adhesion molecules. Fatty acids might therefore be involved in an acute increase in the inflammatory response. Short-term effects of lipid modulation on morbidity have also been shown in the Myocardial Ischemia Reduction with Aggressive Cholesterol Lowering (MIRACL) study [36]. In this multicentre trial, patients with unstable angina pectoris or non-Q-wave myocardial infarction treated with atorvastatine had a lower mortality, lower incidence of recurrent ischemia, non-fatal myocardial infarction and cardiac arrest than placebo-treated controls. Further analysis of these patients suggests that the beneficial effect of this statin therapy might be related to modulation of the cellular inflammatory response [37].

The second important finding of our study was the compartmentalized cytokine production, with levels of IL-6 and IL-10 that were significantly higher in mediastinal fluid samples than in plasma samples. Compartmentalized cytokine production has been demonstrated in animal models [38,39] and several clinical settings [40-42]. Our results clearly demonstrate local production of cytokines in the mediastinal cavity after cardiac surgery. The myocardium is a major source of cytokines. Sampling of blood from the coronary sinus in patients undergoing CABG showed increased levels of TNF-α and IL-6 than in serum [43,44]. In patients with signs of myocardial necrosis the inflammatory response originates from the myocardium itself [45,46].

Compartmentalized cytokine production is also suggested in a study in which elective percutaneous coronary intervention with and without cardiopulmonary bypass and cardiopulmonary bypass supported CABG were compared [47]. Patients undergoing CABG had much higher IL-6 levels than those undergoing the other interventions, suggesting that the local surgical trauma contributes more to the inflammatory response than the systemic reaction to cardiopulmonary bypass. Our data, in concordance with these studies, suggest that the operative trauma to the myocardium and its surrounding tissues is the major source of cytokine production after cardiac surgery. It is unlikely that the systemic inflammatory response syndrome response elicited by the extracorporal bypass has a major role because IL-6 and IL-10 production in the mediastinal cavity exceeded systemic production 1,000-fold and 10-fold, respectively. The local operative trauma and possibly ischemia-reperfusion injury is a more likely explanation for the higher local cytokine concentrations. These results also imply that studies trying to unravel the mechanisms underlying the effect of glucose and insulin on inflammatory processes should preferably take place in the compartment of interest. In this context it would be interesting to study cytokine concentrations in the coronary sinus under various conditions.

Our results are in conflict with several other studies showing that hyperglycemia increases the production of pro-inflammatory cytokines [21,22]. Although differences between glucose levels in the two treatment groups were highly significant, hyperglycemia in the conventionally treated patients was milder than in several other studies and this might have resulted in an impaired trigger for the immune system to change the balance. As a result of modern peri-operative techniques, the systemic inflammatory response syndrome induced by surgery and cardiopulmonary bypass was relatively mild, as is reflected by relatively low APACHE II scores in both groups. In the study by van den Berghe *et al*. [11] the largest reduction in mortality was found in patients with multiple organ failure and sepsis. In septic patients the systemic inflammatory response results in very high concentrations of pro-inflammatory cytokines. It might be that under these circumstances strict glucose control can shift the cytokine balance toward a more anti-inflammatory pattern.

One might speculate that the timing, duration and dosage of insulin could have influenced the results of this study. Instituting insulin therapy before admission to the ICU would probably not have changed the cytokine balance, because median blood glucose levels on admission were within the target range of insulin treatment, and blood glucose levels became different between the groups only after 4 hours of treatment. It is unlikely that insulin treatment beyond the first 24 hours would have influenced the cytokine balance significantly, because after the first 18 hours glucose levels started to normalize in the control group, and cytokine concentrations are known to decline rapidly after the first day of surgery. Because the beneficial effects of post-operative glucose control are most probably related to lowered blood glucose levels rather than the insulin dose, it is not to be expected that increasing levels of insulin will change the inflammatory response [12,13].

## Conclusion

Strict glucose regulation in acute and critically ill diabetic and non-diabetic patients reduces morbidity and mortality in several clinical settings. The exact mechanisms underlying these acute beneficial effects of intensive insulin therapy remain unknown. Our study shows that a change in balance between pro-inflammatory and anti-inflammatory cytokines does not explain the beneficial effects seen in ICU patients after cardiac surgery. The importance of a compartmentalized cytokine production implicates that studies that focus on systemic effects of glucose and insulin should be interpreted with caution.

## Key messages

• 	The beneficial effects of strict glucose control cannot be explained by a change in cytokine balance.

• 	Cytokine production in patients after cardiac surgery is highly compartmentalized.

## Abbreviations

CABG = coronary artery bypass grafting; ICU = intensive care unit; IL = interleukin; NF = nuclear factor; TNF = tumor necrosis factor.

## Competing interests

The authors declare that they have no competing interests.

## Authors' contributions

CH coordinated the clinical study. All authors participated in the design of the study and writing of the manuscript. All authors read and approved the final manuscript.

## Figures and Tables

**Table 1:** Baseline characteristics of patients

Characteristic	Conventional treatment (*n *= 10)	Intensive treatment (*n *= 10)	*p*
Male sex, number (%)	8 (80)	10 (100)	
Age, years	63.2 ± 6.6	65.2 ± 8.7	0.57
Body mass index	26.4 ± 3.1	28.4 ± 4.1	0.23
Duration of operation, minutes	181 ± 52	160 ± 34	0.31
Time on cardiopulmonary bypass, minutes	90 ± 30	83 ± 27	0.57
Blood glucose on admission, mg/dl	96.7 ± 19.6	113.3 ± 14.9	0.05
Parsonnet score	3.0 ± 4.2	5.5 ± 4.2	0.20
APACHE II score	9.2 ± 2.7	9.0 ± 3.4	0.88

Values are means ± SD. APACHE, Acute Physiology and Chronic Health Evaluation.

**Table 2:** Post-operative data

Event	Conventional treatment (*n *= 10)	Intensive treatment (*n *= 10)	*p*
Time between end of surgery and admission to the ICU, minutes	11.4 ± 7.7	18.4 ± 9.1	0.10
Time in ICU, hours	20.3 ± 2.5	22.1 ± 1.8	0.09
Time on ventilator, hours	9.8 ± 4.6	11.2 ± 6.6	0.65
Creatinine kinase, U/l			
On admission	342 ± 371	273 ± 171	0.60
At 16 hours after admission	649 ± 483	684 ± 499	0.88
Erythrocyte transfusion, units	1.67 ± 1.5	1.10 ± 0.88	0.32
Platelet transfusion, units	0.63 ± 0.74	0.33 ± 0.50	0.35

Values are means ± SD. ICU, intensive care unit.
